# Design of a Multipoint Cost-Effective Optical Instrument for Continuous In-Situ Monitoring of Turbidity and Sediment

**DOI:** 10.3390/s20113194

**Published:** 2020-06-04

**Authors:** T. Matos, C. L. Faria, M. S. Martins, Renato Henriques, P. A. Gomes, L. M. Goncalves

**Affiliations:** 1 MEMS-UMinho, University of Minho, Campus de Azurém, 4800-058 Guimarães, Portugal; carlosfaria@dem.uminho.pt (C.L.F.); mmartins@dei.uminho.pt (M.S.M.);; 2 Institute of Earth Sciences, University of Minho Pole, Campus de Gualtar, 4710-057 Braga, Portugal; rhenriques@dct.uminho.pt; 3 Centre of Molecular and Environmental Biology (CBMA), University of Minho, 4710-057 Braga, Portugal; pagomes@bio.uminho.pt

**Keywords:** turbidity sensor, turbidity, sediment processes, suspended sediment, optical sensor, oceanography

## Abstract

A cost-effective optical instrument for continuous in-situ monitoring applications is presented. With a production cost in raw materials of 38 €, a power consumption of 300 µA in sleep mode and 100 mA in active mode (5 ms reading), and a capacity to monitor turbidity and sedimentary displacement at eight different depths in the water column, the sensor was developed for sediment monitoring in coastal areas. Due to the extent and dynamics of the processes involved in these areas, observations require a wide spatial and temporal resolution. Each of the eight monitoring nodes uses one infrared backscatter channel, to estimate turbidity and sediment concentration, and one ultraviolet with one infrared transmitted light channels to distinguish organic/inorganic composition of the suspended material load. An in-lab calibration was conducted, using formazine to correlate turbidity with the electronic outputs of the instrument. An analysis of the influence of external light sources and correction techniques were performed. Moreover, an in-lab experiment was conducted to study the behaviour of the sensor-to-sediment transport, wash load and sediment accumulation. The device was deployed, with a water level sensor, in an estuarine area with high sediment dynamics. The monitoring data were analysed, showing the potential of the device to continuously monitor turbidity, sediment processes, and distinguish between organic and inorganic matter, at the different depths in the water column.

## 1. Introduction

Sediment is a set of naturally occurring particles that are broken down from rocks by weathering and erosion or formed by natural chemical processes or by biological processes. These particles are subsequently transported by the action of wind, water or ice, or by the force of gravity and form deposits when the transportation agents weaken or stop acting [1,2,3]. While the term is often used to indicate rock originated minerals with several dimensions, such as clay, silt and sand, decomposing organic substances and inorganic or biogenic materials are also considered sediment [4]. Sediment materials are typically small, with clay defined as particles less than 0.00195 mm in diameter, and coarse sand reaching up to 2 mm in diameter [5]. However, during floods or other highly energetic events, large rock fragments are also classified as sediments once they are detached from a previous rock formation and are carried downstream. Whenever any of these particles are carried in the course of the water (in liquid or solid-state) or any other moving fluid, such as air (wind), it is called sediment transport.

Sediment transport, or sediment load, is the movement of organic and inorganic particles in a moving mean, such as water [6]. In general, the higher the flow rate, the more sediment can be transported, as well as larger particles. Sediment transportation in an aquatic environment can be strong enough to suspend particles in the water column, as they move downstream, or simply push them along the bottom of the waterway [7]. Sediment transport can be divided into three different processes: bed load, suspended load and wash load. Depending on the characteristics and properties of the streamflow, carried material, and the watershed, the sediment transport can be divided into three different types: bed load, suspended load and wash load [8].

Bedload is the portion of sediment transport that rolls, slides or bounces along the bottom of the waterway [9]. This sediment is not considered suspended, as it is in constant contact with the streambed, and its movement is neither uniform nor continuous. Bedload occurs when the force of the water flow is strong enough to overcome the weight and cohesion of the sediment, making it move [10].

In situations where the flow rate is strong enough, some of the smaller and lighter particles that are settled in the stream floor can be pushed up into the water column and become suspended. The size of the particles that can be carried as the suspended load is dependent on the flow rate (the higher the velocity of the water body, the higher the suspending force applied to the sediment material). Larger and heavier particles are more likely to fall through the upward currents to the bottom, unless the flow rate increases, increasing the turbulence at the streambed. Moreover, suspended sediment will not necessarily remain suspended if the flow rate slows [11].

The wash load is a subset of the suspended load. It is comprised of the finest suspended sediment, typically less than 2 µm in diameter [12]. The wash load differentiates from the suspended load because it will not settle to the bottom of a waterway during a low or no flow period. Instead, these particles remain in permanent suspension as they are small enough to bounce on water molecules and stay afloat.

**Figure 1:**
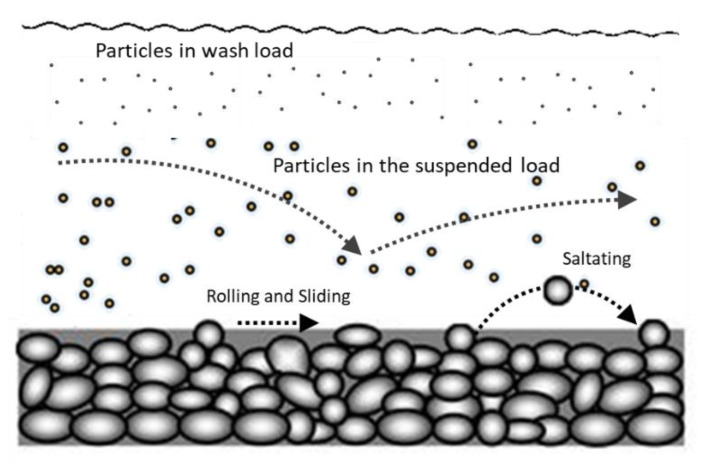
Sketch of sediment transport in water.

As represented in Figure 1, suspended sediment can have different behaviours at different depths along the water column of streamflow. In estuarine and seashore areas, materials are subject not only to the strength of the downstream course but also to the maritime currents, tide and undulation, the different watershed and geological dynamics, seasonal and climatic effects and occurrence of storms, pollution and other anthropic induced processes. For these reasons, sediment processes are difficult to study, model and predict [13], to have proactive management and protection of the coastal areas as their associated societies and ecosystems.

The quantification of sediment transport and deposition in the seaside area is essential to understand the evolution of the littoral coast [14]. However, it has been verified that the correlation between the modelling data and the measurements made in the field is not always satisfactory, mainly due to the great variability and complexity of sedimentary processes, the variation of sea conditions from place to place [15,16] and the lack of more regular observation time series. Therefore, continuous and high spatial resolution monitoring becomes essential for an effective study in each area of action.

This monitoring can only be achieved by autonomous electronic sensors, able to provide reliable data of continuous measurements in the field. The current state of the art of in situ monitoring sensors, to measure sediment processes, relates mainly to acoustic and optical technologies. The acoustic ones, typically referred as Acoustic Backscatters (ABS), use a piezoelectric transducer that emits ultrasonic pulses that are reflected by the suspended material, and one or more ultrasound receivers sense the acoustic echoes, to estimate the amount of sediments, and in some cases, its size [17,18]. The major disadvantage of this technology is its high cost and complexity, compared to the optical technology [19].

The optical sensors aim to measure turbidity, which can provide an indirect estimation of sediment concentration and transport [20]. This type of sensor uses a light source at a certain frequency to illuminate the sample, where the suspended material will absorb and scatter the light, which is measured by optical receivers to provide the turbidity measurement. Due to its ease of use, smaller size, higher energy efficiency and lower cost, the optical turbidity sensors are the most popular instruments to measure sediment/turbidity in situ.

Commercial brands such as Seabird, Seapoint Sensors, Valeport, s::can, among others, offer a wide range of turbidity sensors for in situ purposes. While these instruments offer a good precision, accuracy and the necessary conditions for continuous monitoring, their price is still a challenge for massive deployments (the low-cost series offered are typically above 1200 €). With the challenge of reducing costs to enable the replication of these sensors and to cover a wide spatial resolution, in recent years the scientific community has been dedicated to developing low-cost oceanographic instruments.

Matos et al. [21] presented a low-cost (less than 20 €) turbidity sensor for coastal monitoring. The device was developed to continuously monitoring water parameters in situ, and is integrated in the Next-Sea Project [22,23,24], which has the main philosophy of developing cutting edge technology that is low-cost, for massive replication and deployments, low-power, to extend the lifetime of the batteries and reduce maintenance needs, low-size and light, for ease of use and installation, with no need of big ships or cranes, and fully submersible, to avoid floater restrictions. The previously developed sensor, referred to hereinafter as the SPM Sensor, is an optical single-point device that measures turbidity (in NTU or another unit, provided that calibration is established), suspended particle matter concentration (in g/L) and distinguishes between organic and inorganic suspended material (also in g/L).

The presented manuscript presents an innovative device that takes advantage of the technology used in the SPM Sensor, but allows to monitor turbidity and sediment processes at different depths in the water column. While the vertical replication of the SPM Sensor could be an approach, the newly developed instrument presents advantages in ease of use and installation, energetic efficiency and cost.

## 2. Sensor Design

The developed instrument is a continuation of the previously SPM Sensor [21] that uses three light detection techniques to measure turbidity, sediment concentration and distinguishes between organic/inorganic sediment. The SPM Sensor was developed in a radial configuration, using one infrared backscattering channel (optical receiver placed at 135° related to the emitter), one infrared nephelometric channel (optical receiver placed at 90° related to the emitter), and one ultraviolet and one infrared transmitted light channels (optical receiver placed at 0° related to the emitter). The use of multiples angles to measure turbidity allows a wide dynamic range and precision [25].

The backscattering technique relates to the measurement of scattered light by the suspended material [26]. It offers a wide range of measurement and it is the most popular technique used in commercial turbidity sensors (typically referred to as Optical Backscatter Point Sensors—OBS). On the other hand, the nephelometric technique, which also measures scatter and diffuse light but at a different angle, is typically used for high-accuracy and low-turbidity values and is mostly used for inline sensors on wastewater and water treatment plants [27,28]. Finally, the transmitted channels measure the attenuation of light in a straight path, by the scattering and absorption caused by suspended material. This technique is not very popular to measure turbidity, due to its high sensitivity to particle size, shape and colour. For the SPM Sensor, it was used to distinguish between organic and inorganic matter, taking advantage of the different responses of matter to different wavelengths (ultraviolet and infrared) [29].

SPM Sensor results showed that while to have a wide dynamic range to measure turbidity the backscattering, nephelometric and transmitted light techniques should be used, all three techniques have demonstrated good performance as standalone sensing systems. For this reason, a new design was adopted for the developed instrument.

**Figure 2:**
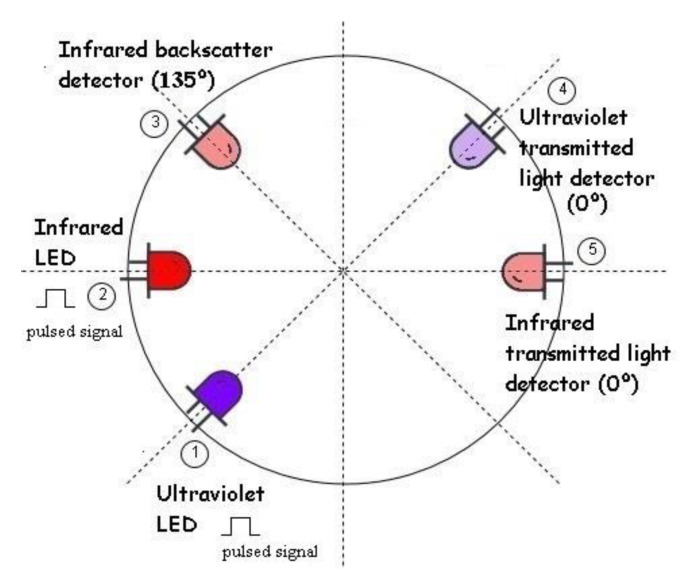
Design of the sensor. It uses two infrared channels: one IR LED (2) and two transducers that measure optical backscattering (3) and transmitted light (5); and one ultraviolet channel: UV emitter (1) and wideband receiver (4), adapted from [21].

The ultraviolet and infrared transmitted light channels were maintained to distinguish between organic and inorganic matter. For turbidity and sediment concentration, the new device only uses the backscatter technique. Since the nephelometric technique has a high performance for low turbidity values that are not expected in coastal areas, this light detection technique was not used (see Figure 2).

**Figure 3:**
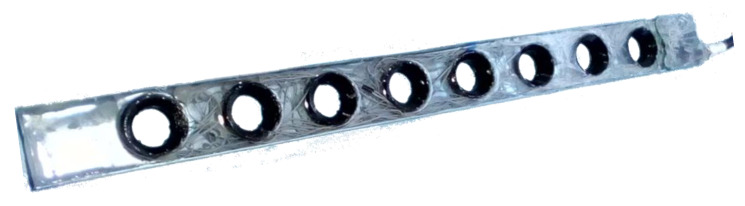
The developed instrument comprises 8 monitoring nodes, with the backscatter and transmitted light channels, along a 645 mm bar, to be placed vertically in the water column (the instrument is presented in this figure in the horizontal).

The developed new instrument integrates several sensing nodes along a one-body and compact structure controlled by a single microprocessor. The developed device is a 645 mm × 55 mm × 15 mm bar, with eight measuring nodes displaced vertically by 70 mm from each other (see Figure 3). Each node has a 30-mm diameter and comprises the optical transducers presented in Figure 2. The instrument was built in a scale and modular philosophy, so that similar bars can be fixed on the top of the previous one (from stream bottom to surface) and increase the number of nodes and depth monitoring along the water column. When using more than one bar, all of them are serial connect to pass information from bottom to top, and all the connected bars will be recognized as a single longer bar.

The structural housing of the sensor was manufactured with epoxy, a resin material to comprise the electronic printed circuit board (PCB) and to meet the watertight needs for full submersion. This structure, installed vertically and along the water column, allows to perform the same measurements as the previous developed SPM Sensor, but at different depths and using a single apparatus, instead of using multiple sensors.

One objective of the new sensor development was for it to be low-cost to allow massive replication. While the SPM Sensor has a production cost of 20 €, in raw materials, and performs a single point measurement, the new device has a production cost of 38 € and take measurements at eight different levels of depth.

### Hardware

In the hardware, changes were made concerning the previous SPM Sensor. For the optical transducers, while T1-3/4 (5 mm) packages were used previously, for this new device, T1 (3 mm) transducers were used to minimize its size. For the infrared channels, the light-emitting diode (LED) TSUS4300 (950 nm, 16° emitting angle and 18 mW/sr radiant intensity at 100 mA) and the phototransistor TEFT4300 (950 nm, view angle of 30° and 1 nA dark current) were selected, see Figure 2. For the ultraviolet pair, the LED UV3TZ-390-30 (390 nm, 15° emitting angle and 10 mW emitting power at 15 mA) and the wideband phototransistor SFH 3310 (spectral range of sensitivity from 350 to 900 nm, view angle of 75° and 3 nA dark current) were used. Notice that the use of LEDs was maintained, instead of LASERs, due to their good performance in SPM Sensor, wide commercial offer and lower price.

For efficient management of all nodes and respective optical emitters and receivers, a microprocessor was carefully chosen. The smt32l496zg from the stm32 L4 family was selected due to its low power, 24 × 12-bit ADC channels (3 photodetector analogic signals × 8 measuring nodes requires 24 ADC channels) and several GPIOs (8 IR LEDs and 8 UV LEDs to be controlled). For a different approach, a simpler microprocessor could be used complemented with a multiplexer; however, the sensitivity of the instrument could be affected due to the internal resistance of the multiplexer and it would slightly increase the cost and power consumption of the sensor.

**Figure 4:**
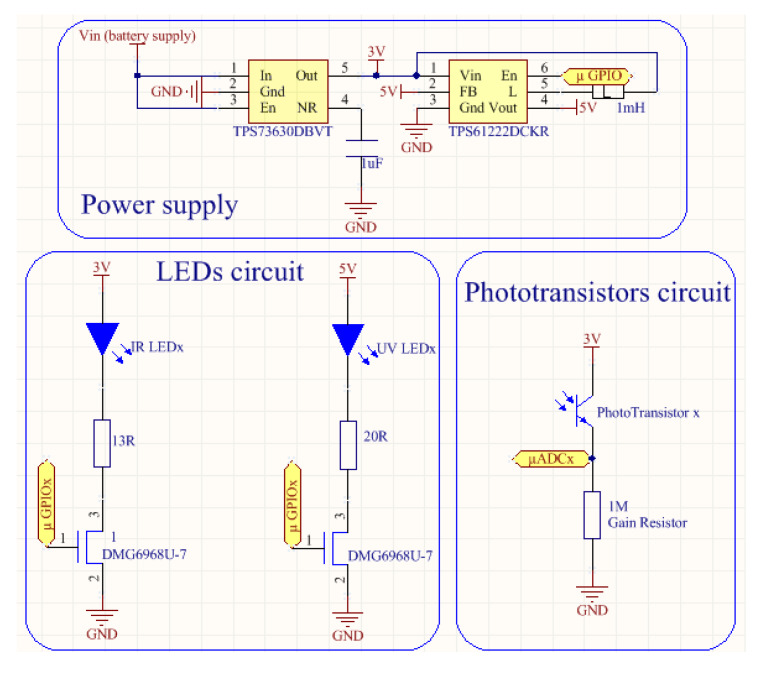
Schematics of power supply (**top section**), LEDs circuit (**bottom left section**) and phototransistors circuit (**bottom right section**).

The sensor was intended to be supplied by a common 3.6 V Li-Ion battery. For the power supply circuit, the TPS73630DBVT low-dropout regulator (3 V fixed output, 400 mA max current, 75 mV dropout voltage and 200 uA quiescent current) was used, as presented in Figure 4. While the whole system works with 3 V, the UV LED UV3TZ-390-30 has a forward voltage of 3.4 V so a higher supply is needed. For this purpose, the TPS61222DCKR boost convertor was used to supply the UV LEDs circuit (95% efficiency, 5.5 uA quiescent current and 200 mA switching current). Voltage regulators were used since constant voltage is mandatory to minimise LED intensity variations.

The LED circuits are controlled by the microprocessor GPIOs that switches a low-on resistance N-channel MOSFET (≈30 mΩ) to turn them on and off. The phototransistor instrumentation circuited was designed with a simple current-to-voltage converter using a gain resistor. The resistor voltage potential is read by de microprocessor ADCs to be processed (Figure 4).

For the communications, two low power RS485 drivers (LTC1480CS8) were used for two UARTs of the microprocessor. These two communication channels were designed by anticipating the modular integration of multiple bars along the water column, allowing to work as a single longer bar. One RS385 bus was used to connect to the bar above, and the other to the under one.

The developed hardware has a power consumption of approximately 100 mA during the readings (5 ms) and 300 µA in sleep mode. This means that using a common mobile-phone 3000 mA × 3.6 V lithium battery, the sensor has an autonomy of more than 1 year to take continuous measurements at a 1/min sample rate.

## 3. In-Lab Calibration

To provide reliable data in situ measurements, the sensors must be calibrated. In the previous work [21], the SPM Sensor was calibrated with formazine (NTU), seashore sand (concentration of suspended particles in g/L) and organic matter using a phytoplankton solution (to demonstrate the efficiency in distinguishing organic from inorganic material).

For this new device, and since the major objective is the measurement of sediment circulation at different depths in the water column, a calibration with seashore sand should be used. However, in past experiments calibrating the SPM Sensor, to get a homogeneous solution and keep the particles in suspension, a mixer needed to be used. For this new device and due to its “long” size, it was unpractical to use the same experimental design to calibrate the sensing nodes. The alternative was to perform the calibration only with formazine, which is a homogeneous solution, and no mixer was needed. Despite turbidity units and suspended particles concentration are not interchangeable, a change in turbidity is directly related to a change in suspended particle concentration.

### 3.1. Turbidity Calibration (NTU)

To perform the turbidity calibration, the procedure described in [21] was used. Starting with a commercial 4000NTU Formazine Standard solution, the sample was diluted in deionized water following the methods and procedures of the Hach Water Analysis Guide [30]:

(1) $$Dilution_{factor}=\frac{volume_{total}}{volume_{\;sample}}=\;\frac{volume_{\;deionized\_water\;}+volume_{\;NTU\_sample}}{volume_{\;NTU\_sample}}$$

For each calibration sample, 10 measurements were recorded for each photodetector (backscatter, IR transmitted, and UV transmitted) for every eight nodes. The sensor performed with a high accuracy, with a major reading error of 2 mV. This value is related to the ADC resolution.

**Figure 5:**
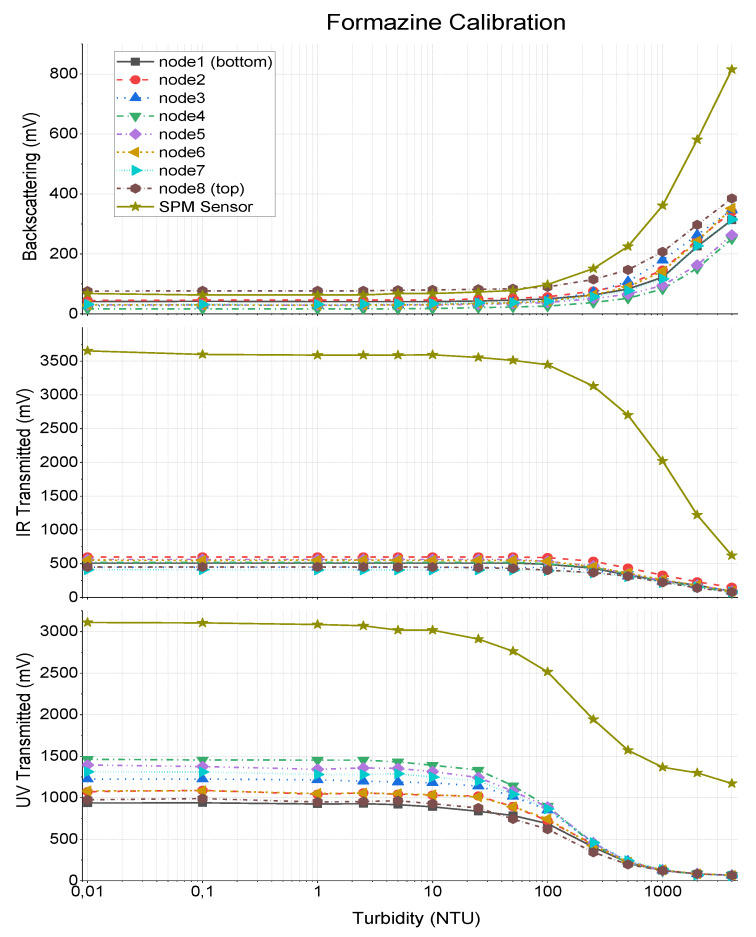
Formazine calibration results for the three light detection techniques of each of the 8 nodes and comparison with SPM Sensor curves.

Figure 5 shows the calibration results of the developed device, as well as the previous calibration curves of the SPM Sensor. The behaviour of the different techniques was as expected, according to the theory presented in SPM Sensor Manuscript [21].

In the backscattering technique, for the maximum turbidity value (4000NTU), the maximum optical scattering by suspended particles was achieved, similarly to the photodetector, which results in a higher electrical output. As the sample is diluted, the number of suspended particles decreases, as the optical scattering and electrical output of the backscatter photodetector.

For the transmitted light techniques (both infrared and ultraviolet) the opposite happens. For the 4000NTU solution, the emitting light is highly reflected and absorbed in its direct path, so the sensed light by the photodetector is minimum. As the turbidity decreases, the passage of light increases as the electric output of the receiver transducer. The output voltages of the sensors, as a function of turbidity of the sample solution, is presented in Figure 5. The output voltages obtained in the three measuring techniques (IR backscattering, IR transmission, and UV transmission) are presented in three separate graphs. Each graph presents the output voltage of the eight nodes of the sediment bar, from top to bottom node, as well as the output voltage of the previously developed SPM sensor [21].

Comparing the curves of the developed instrument with the SPM Sensor, the new device presents a lower sensitivity, mainly in IR and UV transmitted channels. This happened due to the change for smaller transducers (3 mm in the step of 5 mm used in SPM Sensor), since the new emitters have less radiant power and photodetectors less sensitivity. Note that the gain resistor used was the same for both devices (1 MΩ in Figure 4). It is important to notice that both transducers and gain resistors can be easily changed during the manufacture of a new sensor.

The developed device can detect changes in turbidity above 10NTU, which underperforms against the SPM Sensor, but is still adequate to the coastal areas where higher turbidity values are expected (as an example, water for human consumption are limited to 5NTU, which is clear water). At the upper limits of the scale, the backscattering sensor can measure higher turbidity values, since only 200–400 mV were obtained with 4000NTU.

The calibration values from Figure 5 are used to feed a linearization table in the microprocessor of the sensor, used to calculate the turbidity in real time from the sensors output voltage during in-situ deployments.

### 3.2. External Light Calibration

Previous calibrations with the SPM Sensor showed that this type of optical device is influenced by the external light. This is extremely important for in situ monitoring, as the solar light can produce interferences in the measurements.

**Figure 6:**
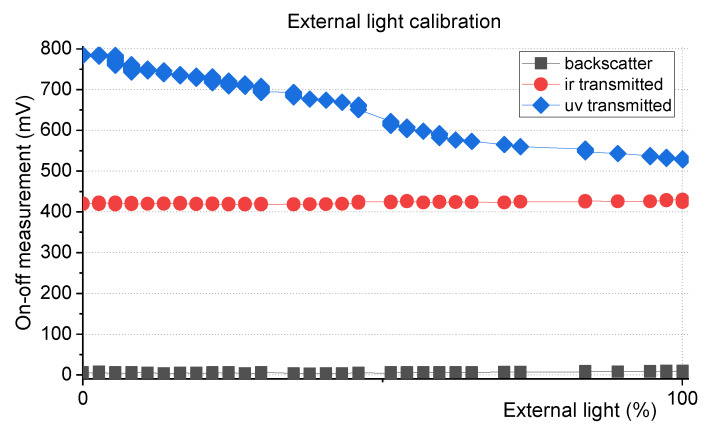
External light calibration results. The device was submerged in a sample of water and exposed to an external light source while measurements with LEDs on and off were recorded.

To reduce the external light interference, the instrument was submerged in a water solution and exposed to an external light source, while measurements with the LEDs on and off were recorded. The graph in Figure 6 represents the difference between the voltage obtained when LEDs are turned on, and the voltage obtained when the LEDs are turned off, in the three proposed sensing technologies, when ambient light changes from dark (0%) to a predefined value of ambient light is a sunny day (100%).

Since the instrument was submerged in a sample with constant turbidity, an increase of the electrical voltage output of the photodetectors (both for LEDs on and off measurements) is expected with the increase of the power luminosity of the external light. In this instrument (since the emitters/receivers pairs have lower sensitivity than previous SPM instrument), for backscatter and transmitted infrared channels, the difference between the output voltage with the LEDs on and the LEDs off remained constant, when ambient light changes from 0% to 100% (see Figure 6). This means that this difference (also called ON-OFF technique) can be used to measure turbidity, avoiding the interference of ambient light. However, the same did not happen with the ultraviolet transmitted channel, and calibration of the sensor as a function of ambient light is still needed.

As for the SPM Sensor [21], a mathematical expression was calculated to eliminate the external light effect, with $correction_{factor}$ corresponding to the photodetector correction factor and $off_{value}$ the voltage measurement of the external light influence (measurement with IR LEDs off). The on-off measurement of the UV channel will be divided by this factor.

(2) $$correction_{factor}=0.0000002*off_{value}{}^{2}-0.005*off_{value}+1.0174$$

**Figure 7:**
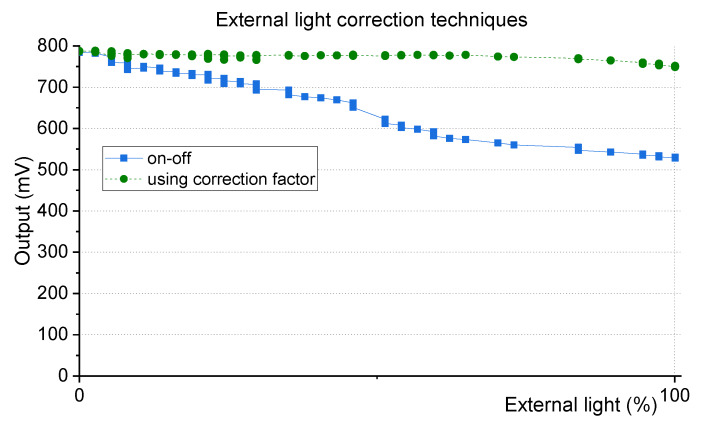
Demonstration of the effectiveness of the developed technique to correct external light influence. The blue squares and the straight line show the on-off measurement curve of Figure 6. In green squares and dashed line, the on-off measurement curve but with the correction factor of (2) applied.

Using the mathematical equation above, the turbidity measurements can be corrected as shown in Figure 7.The equations are used to process the in-situ data and to eliminate the offset caused by any kind of external light. The factor is applied to the electrical on-off value that afterwards is used to estimate the turbidity value using the linearization table of the NTU calibration.

## 4. In-Lab Evaluation with Seashore Sand

**Figure 8:**
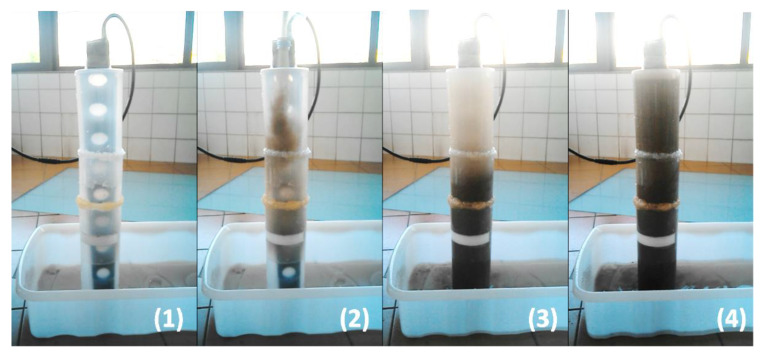
In-lab experiment design. The sensor was submerged in a container with deionized water (**1**) and seashores sand was released from the top (**2**). The release was repeated (**3**) until the container became saturated with sand (**4**).

To test the behaviour of the developed instrument, an in-lab evaluation was performed with seashore sand, collected from the local where the device was intended to be deployed. The instrument was submerged in a container full of deionized water (minimum turbidity value), and seashore sand was gradually released from the top until it settles. This movement was repeated until the container became saturated with sand (see Figure 8).

**Figure 9:**
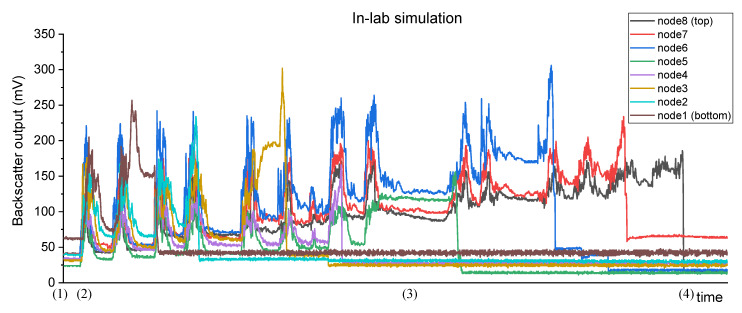
Electrical output record of the backscatter channels of the 8 nodes during the in-lab simulation with seashore sand.

During the experiment, the electrical output of the photodetectors of all nodes was recorded with a sample period of 200 ms. The graph in Figure 9 shows the measurement of the backscatter channels of the eight nodes of the sensor.

At the beginning of the experiment (time (1)), the photodetectors presented an electrical output corresponding to a low turbidity value. Whenever the sand was released (e.g., time (2)), all nodes presented a peak in its output and then it converged slightly above to the previous value (this happens because of the passage of the particles that are settling from the top to the bottom of the container). While this does not faithfully simulate the transport load in situ, it shows that the sensor can measure abrupt changes in turbidity due to the sediment transport.

As the sand was released, while the bigger particles settled in the bottom, the smaller and lighter ones remained suspended, bouncing in the water (wash load) and increasing the turbidity (e.g., time (3)). This behaviour was detected by the photodetectors and it was reflected in the constant increase of its electrical output (the mean value is continuously increasing during the test).

Lastly, while it was not its main purpose, the device showed the potential to also monitor sediment accumulation. With the deposition of the released sand in the bottom of the container, its accumulation would incrementally burry all eight nodes. Analysing the graphic, the backscatter from node1 is the first to abruptly change its output to a very low value (when the node is buried, the photodetector can no longer receive light from the LED since the sediments act as an opaque wall), followed by nodes 2 to 8.

It is important to notice that the electrical output of each photodetector for the lowest turbidity value is higher than when the node is buried (dark current of the photodetector), so a low turbidity value is not confused with a buried node. In the same way, if the top nodes of the instrument are out of the water, the measurement will not be confused with low turbidity levels. For this case, and since the light attenuation in water is higher than in the air, the sensor would produce high electrical output values, corresponding to negative turbidity values, so a threshold can be defined.

## 5. In Situ Monitoring

The device was installed, from 10 April 2019 to 16 April 2019, in the Cávado river mouth, Esposende—Portugal (41°31′56.6″ N 8°47′04.8″ W). This location was chosen due to its high sedimentary dynamic, where the formation of shallows is constant, and the geomorphology of the area is continually changing. With a high dynamic in the sedimentary processes, it is expected to also detect changes in turbidity.

The instrument was connected to a data-logger that received and stored the monitoring information with a sample rate of 1 min. The developed device was buried in the bed stream, with nodes 8, 7 and 6 uncovered, and the remaining nodes buried in the bottom sand. Complementarily, a water level sensor was used to obtain the height of the water column by measuring the pressure from bottom to top and detect water level fluctuations due to tide cycles. These data were used to correlate the turbidity measurements with the hydraulic dynamics of the zone.

**Figure 10:**
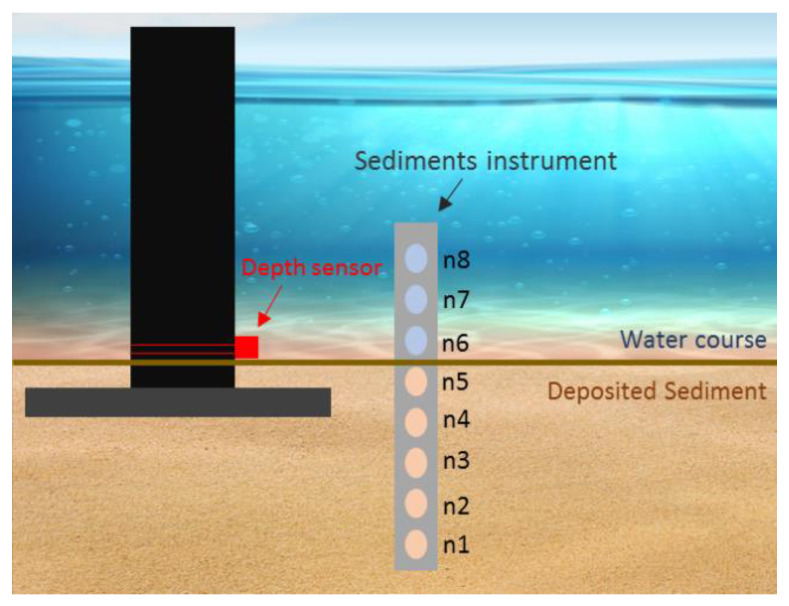
In-situ installation design. The depth sensor was deployed at the bottom of the watercourse, with the zero-depth matching the middle of nodes 5 and 6 of the sediment sensor.

The water depth sensor was attached to an infrastructure, in a fixed position, with the zero-depth arbitrary defined as the bottom of the watercourse at the moment of the installation—the pressure sensor position—and corresponding to the middle of nodes 5 and 6 of the sediments sensor, as Figure 10 shows. This setup was optimized only for calibration and field test purposes, without aiming to obtain datum based values of water or sedimentation depths. In further monitoring studies with the developed instrument, the top position of the sediment sensor will be collected with the Global Navigation Satellite System (GNSS) and calibrated considering the measurement of data from the national geodetic network. The same procedure will be used for positioning the pressure depth sensor, which will migrate to the top of the bar. This way, absolute datum-based heights will be obtained.

This configuration would allow to measure turbidity at three different depths (nodes 6, 7 and 8) and detect changes in the deposited sediment. If node 5 was uncovered, streambed material was being transported (scour), otherwise, if node 6 was covered, sedimentary material was being deposited in the streambed (sediment accumulation). Finally, even that the calibration for the distinguish between organic/inorganic material has not been established, a demonstration of the different responses of the matter to the ultraviolet and infrared transmitted light channels is presented.

### 5.1. Turbidity Monitoring

**Figure 11:**
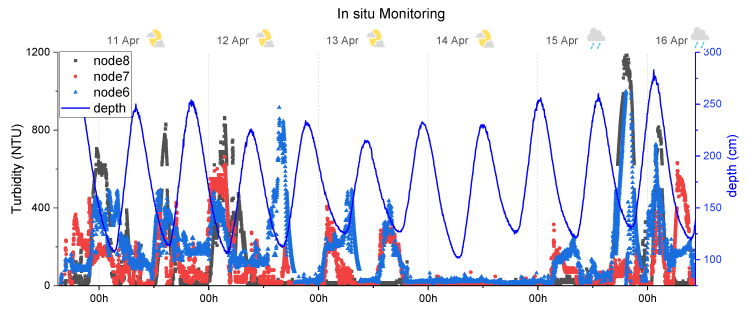
In-situ monitoring results in the Cávado river mouth from 10 April to 15 April 2019. The grey squares, red circles and blue triangles show the measurement data of nodes 8, 7 and 6, respectively. The blue line shows the measurement of the water level.

The graph in Figure 11 shows the measured turbidity (NTU) of the three unburied sensor nodes, during the 6-day test. The depth of the water (resulting from tidal oscillations) is represented, in the right-side axis. At the top of Figure 10, the weather is also represented with pictograms. The results show a pattern between the tide cycles and the turbidity measurement. The maximum turbidity values were detected during the low tide, and its values decreased with the increase of the tide. This behaviour can be explained by the higher turbidity that originated from the river stream when compared to the cleaner water coming from the sea, which is typical in a river mouth. Moreover, when the tide is high, the water volume in the estuary increases, so the suspended material is more diluted, which leads to lower turbidity values.

A curious behaviour occurred during this deployment. The monitoring was performed during the neap tide, a natural event that occurs when the sun and moon are at right angles of each other. This produces moderate tides, meaning that high tides are lower and low tides are higher than average and that the turbulence caused in the estuary stream is lower than the normal. The neap tide was reflected in the monitoring results of the sensor, during 13 and 14 April, when the medium variation of depth was lower (see the depth curve in Figure 11 and compare its amplitude during the different days). The turbidity peaks during the low tides on 13th April are lower than for the days before. On 14 April, no peaks were detected at all, and the turbidity remained on average at 27NTU, 32NTU and 35NTU from node 8, 7 and 6, respectively. This slight difference in the turbidity values can be explained by the depth difference between the nodes (remember the settling effects of particles in the wash, suspended and bedload), by some inaccuracy in the calibration values (considering the turbidity steps used during the formazine calibration, a difference of 8NTU is acceptable) or even by biofouling interference in the reading. After the neap tide (15 and 16 April) and with the increase of the tide harmonic, the turbidity peaks appeared once again.

For the attempt to measure sediment accumulation, the node 6 was always providing turbidity measuring, which means that it was uncovered during all the experiments. In the other way, the nodes 5 to 1 presented a constant value during the test, above the threshold value, which indicates that the photodetectors are buried. This does not necessarily mean that there were no changes in the deposited sediment, but that the distance of 70 mm between the nodes was not enough to detect any change during the experiment.

### 5.2. Dsitinguish between Organic/Inorganic Matter

In the previous manuscript of the SPM Sensor development [21], a calibration to demonstrate the potential of the sensor to distinguish organic and inorganic suspended material was conducted. The major problem associated with this measurement is that organic material can come in various forms and produce different light attenuation and scatter at the different wavelengths used. To solve this problem, and as future work, we advised to perform a calibration with collected organic suspended material from the locality where intended to deploy the sensor, to have a precise estimation.

**Figure 12:**
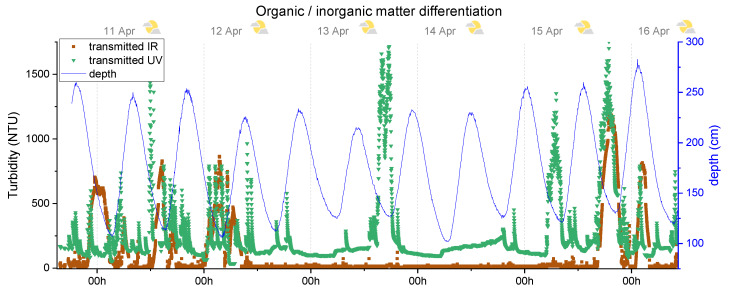
In situ monitoring results for the distinguish between organic/inorganic material, using the ultraviolet and infrared transmitted light channels. The brown squares show the data of IR channel and the green triangles show the data for UV channel, both from node 8. The blue line shows the measurement of the water level.

However, even with the typical calibration with formazine, the developed device presents a similar behaviour of the SPM Sensor to distinguish between organic and inorganic sediment. Figure 12 shows the measured data of ultraviolet and infrared transmitted light detectors of node 8.

Upon observing the resulting data, is can be noted that the ultraviolet detector line produced higher turbidity values that the infrared one. These results are in agreement with the theory of the higher absorption of organic matter to ultraviolet wavelength when compared to the infrared, which results in a lower power luminosity sensed by the UV transmitted light channel and in a higher turbidity output. This difference between the IR and UV turbidity values occurs due to the existence of organic suspended sediments, and this differential value can be used to estimate the amount of organic matter if an appropriated calibration is established. Since the estuary area is the end of the river course and the deployment was conducted in an urban area, a high organic load in the locality is always expected. This particular behaviour was also demonstrated in a similar experiment with the SPM Sensor [31].

Analysing the monitoring information, the higher turbidity peaks were detected during the low tide, as for turbidity backscatter measurement, and as it should, the infrared transmitted channel is also compliant with the backscatter curves presented in Figure 11. The ultraviolet channel presented similarities with the infrared transmitted channel during these peaks; however, it also shows differences during the neap tide, with the peaks at 13 and 15 April, only detected by the UV channel and not by the IR ones. This behaviour is translated into a high organic load that does not produce significant changes in the water turbidity measured in IR and can be explained by algae blooms or anthropogenic contaminants like pesticides.

While some of the data provided by the sensor can be explained by the factors mentioned before, it is important to note that other events may have contributed to the value of the measurements. Weather events (on 15 April, the anomalous high turbidity peak was probably caused by the rainwater that has dragged dirt by the urban area), wastewater discharges (existence of wastewater pipelines upstream where the sensor was installed), attachment of undesired debris, transported by the stream, biological material in the sensor (biofouling) and the own sediment dynamic in the unknown area, may have also influenced the results.

## 6. Conclusions

A low-cost (38 € production cost in raw materials) and low-power (300 µA in sleep mode) optical oceanographic instrument was developed for in situ continuous monitoring. The devices take advantage of the technology previously developed in an optical turbidity single-point sensor [21], using infrared backscattering and infrared and ultraviolet transmitted light techniques, in a radial configuration, that allows to estimate the turbidity and suspended sediment concentration (depending on the calibration used) and distinguish between organic/inorganic suspended mater, at eight different depths in the water body.

To prepare the instrument for the in-situ deployment, an in-lab calibration with formazine was conducted to establish the correlation between the turbidity and the electrical output of the three channels and compare its performance with the single-point sensor developed previously. The new device presented lower sensitivity and dynamic range than the previous one, due to the replacement of 5 mm optical transducers by 3 mm ones. However, this is easily overcome in further manufacturing increasing the resistor gain in the electronic hardware, or back to use 5 mm transducers packages. Moreover, an in-lab experiment was designed using seashore sand, to demonstrate the sediment processes that could occur during the in-field tests, namely sediment transport, sediment wash load and sediment deposition.

The instrument was deployed for a week in a critical area of sediment dynamics. The monitoring data results show the potential of the sensor to measure turbidity and sediment processes in a continuously monitoring, and at different depths in the water column. The information from a water level sensor was used to correlate its data to the developed sensor and allowed to detected turbidity changes with the tidal cycles and differences in the sediment dynamics behaviour during the neap tide. Even without calibration, the differentiation of organic and inorganic suspended matter estimation was also addressed, and changes in the organic and inorganic loads were demonstrated and how to differentiate them.

It was proposed during the in-lab experiment that the sensor would be able to detect changes in the streambed deposited sediment. During the in-situ experiment, the device was strategically installed to detect changes both in turbidity as in sediment accumulation. Sediment accumulation was insufficient to be measured, considering the large distance between the sensing nodes (70 mm). Therefore, it is recommended to develop a new device focused to monitor sediment deposition with higher resolution.

Efficient monitoring information about sediment dynamics in the coastal areas, to be used to feed the mathematical models, can only be achieved using electronic instruments to provide reliable data and with a wide spatial and temporal resolution. The main objective of this work was to develop an innovative instrument to monitor sediment processes at different depths, to present a low-cost, low-power, and less complex alternative to the vertical replication along the water column of multiple single-point turbidity sensors. The use of the water level sensor during the in-field experiment was very important to understand the sediment transport behaviour with the hydraulic conditions. We recommend that these type of monitoring studies do not focus only on the use of turbidity sensors, but also in the other water parameters that may affect the sediment processes.

Further optimization of the developed device should include an effective calibration with organic matter to not only detect changes in the organic and inorganic load, as demonstrated during the deployment, but also to estimate its concentration in g/L. Organic matter can come in many and different origins, that originate different absorption ratio in both ultraviolet and infrared wavelengths, so we recommend the use of matter collected directly from the local were intended to deploy the instrument. Moreover, an efficient design must be developed to calibrate the nodes with suspended seashore sand, to provide an in-field estimation of not only turbidity (formazine calibration) but also suspended sediment concentration, also in g/L.

Finally, biofouling interference was not detected during the measurements. This can be explained with the use of the 3 mm transducers package (small surface exposed to the water) and, depending on the biological condition of the local, the deployment time could also be insufficient to detect errors caused by the surface fouling in the LEDs and phototransistors. However, it is known that the optical devices are susceptible to biofouling and present errors in long time deployments without maintenance. This is a current problem for the scientific community that, in the last decades, has addressed multiple techniques to try to solve this issue. Further manufacturing of the developed device should consider the state of art in this area to provide reliable data with a high temporal resolution.

## References

[1] Langland M., Cronin T. (2003). A Summary Report of Sediment Processes in Chesapeake Bay and Watershed.

[2] Wang P., Li Q., Li C.-F. (2014). Sedimentology. Developments in Marine Geology.

[3] Aslan A. (2013). Sediments. Encyclopedia of Quaternary Science: Second Edition.

[4] Wetzel R.G. (2001). Limnology: Lake and River Ecosystems.

[5] Duller R.A., Whittaker A.C., Fedele J.J., Whitchurch A.L., Springett J., Smithells R., Fordyce S., Allen P.A. (2010). From grain size to tectonics. J. Geophys. Res. Earth Surf..

[6] Van Rijn L.C. (1993). Principles of Sediment Transport in Rivers, Estuaries and Coastal Seas.

[7] Fredsøe J. (2012). Sediment transport. Handbook of Environmental Fluid Dynamics, Volume One: Overview and Fundamentals.

[8] Dey S. (2014). Bed-Load Transport. Fluvial Hydrodynamics. GeoPlanet: Earth and Planetary Sciences.

[9] Van Rijn L.C. (1984). Sediment transport, Part I: Bed load transport. J. Hydraul. Eng..

[10] Meyer-Peter E., Müller R. (1948). Formulas for Bed-Load Transport. IAHSR 2nd Meeting, Stockholm, Appendix 2.

[11] Van Rijn L.C. (1984). Sediment transport, Part II: Suspended load transport. J. Hydraul. Eng..

[12] Woo H.S., Julien P.Y., Richardson E.V. (1986). Washload and fine sediment load. J. Hydraul. Eng..

[13] Xue Z., He R., Liu J.P., Warner J.C. (2012). Modeling transport and deposition of the Mekong River sediment. Cont. Shelf Res..

[14] Reise K. (2002). Sediment mediated species interactions in coastal waters. J. Sea Res..

[15] Amoudry L.O., Souza A.J. (2011). Deterministic coastal morphological and sediment transport modeling: A review and discussion. Rev. Geophys..

[16] Lesser G.R., Roelvink J.A., van Kester J.A.T.M., Stelling G.S. (2004). Development and validation of a three-dimensional morphological model. Coast. Eng..

[17] Kim Y.H., Voulgaris G. (2003). Estimation of supsended sediment concentration in estuarine environmetns using Acoustic Backscatter from an ADCP. Proc. Coast. Sediment..

[18] Crawford A.M., Hay A.E. (1993). Determining suspended sand size and concentration from multifrequency acoustic backscatter. J. Acoust. Soc. Am..

[19] Gartner J.W. Estimation of Suspended Solids Concentrations Based on Acoustic Backscatter Intensity: Theoretical Background. Proceedings of the Turbidity and Other Sediment Surrogates Workshop.

[20] Davies-Colley R.J., Smith D.G. (2001). Turbidity, suspended sediment, and water clarity: A review. J. Am. Water Resour. Assoc..

[21] Matos T., Faria C.L., Martins M.S., Henriques R., Gomes P.A., Goncalves L.M. (2019). Development of a Cost-Effective Optical Sensor for Continuous Monitoring of Turbidity and Suspended Particulate Matter in Marine Environment. Sensors.

[22] Baptista J.P., Matos T., Lopes S.F., Faria C.L., Magalhaes V.H., Vieira E.M.F., Martins M.S., Goncalves L.M., Brito F. A four-probe salinity sensor optimized for long-term autonomous marine deployments. Proceedings of the OCEANS 2019.

[23] Faria C.L., Martins M.S., Lima R.A., Goncalves L.M., Matos T. Optimization of an Electromagnetic Generator for Underwater Energy Harvester. Proceedings of the OCEANS 2019.

[24] Martins M.S., Faria C.L., Matos T., Goncalves L.M., Silva A., Jesus S.M., Cruz N. Performance evaluation of a PVDF hydrophone for deep sea applications. Proceedings of the OCEANS 2019.

[25] Ronald J., Zaneveld V., Spinrad R.W., Bartz R. Optical Properties Of Turbidity Standards. Proceedings of the Ocean Optics VI.

[26] Bin Omar A.F., Bin MatJafri M.Z. (2009). Turbidimeter design and analysis: A review on optical fiber sensors for the measurement of water turbidity. Sensors.

[27] Khouja H. (2010). Turbidimetry & Nephelometry. Cell Biochem. Funct..

[28] US EPA (1993). Method 180.1: Determination of Turbidity by Nephelometry.

[29] Snyder W.A., Arnone R., Davis C.O., Goode W., Gould R.W., Ladner S., Lamela G., Rhea W.J., Stavn R., Sydor M. (2008). Optical scattering and backscattering by organic and inorganic particulates in U.S. coastal waters. Appl. Opt..

[30] Hach (2013). Water Analysis Guide 09/2013.

[31] Matos T., Faria C.L., Martins M., Henriques R., Goncalves L. Optical device for in situ monitoring of suspended particulate matter and organic/inorganic distinguish. Proceedings of the OCEANS 2019.

